# Supplementary material for: Attentional guidance through object associations in visual cortex
Source: Sci Adv. Author manuscript; Available in PMC 2025 Jan 22. (PMC11468920; doi:10.1126/sciadv.ado6226)
Supplement: Supplementary Material [file EMS202163-supplement-Supplementary_Material.pdf]

Science Advances
AAAS

Supplementary Materials for

Attentional guidance through object associations in visual cortex

Maëlle Lerebourg *et al.*

Corresponding author: Maëlle Lerebourg, m.lerebourg@donders.ru.nl

Sci. Adv. **10**, eado6226 (2024)
DOI: 10.1126/sciadv.ado6226

This PDF file includes:

Supplementary Text
Figs. S1 to S4

Supplementary Text

Behavioral analyses

Overall behavioral accuracy in the search task was 72.46% (sd 8.91). Hit rate was 66.16% (sd 12.61), with 21.16% (sd 10.89) false alarms. Sensitivity (*d'*) was 1.51 (sd 0.61) and criterion (*c*) 0.21 (sd 0.24) on average.

Sensitivity (*d'*) was 1.51 (sd 0.61) and criterion (*c*) 0.21 (sd 0.24) on average. Thus, as intended, finding the targets in the search task was challenging, but not impossible.

We established whether performance was matched for both targets and scene contexts, using a Target (Book, Bowl) x Scene Context (yellow, blue living room) ANOVA. Participants were equally accurate for both targets and both rooms (no main effect of Target: $F(1,33) = 1.04$, $p = 0.32$, $\eta_p^2 = 0.03$; no main effect of Scene context: $F(1,33) = 0.07$, $p = 0.79$, $\eta_p^2 < 0.01$; no Target x Scene context interaction: $F(1,33) = 3.06$, $p = 0.07$, $\eta_p^2 = 0.09$). Next, we tested whether performance differed for search on the two anchor tables, using an Anchor (Table 1, Table 2) x Scene Context (yellow, blue living room) ANOVA. Participants were more accurate on the smaller and brighter table ($F(1,33) = 12.85$, $p < 0.01$, $\eta_p^2 = 0.28$), possibly due to the better contrast between the table and the objects placed on it. However, this advantage depended on the context, as indicated by a significant Anchor x Room interaction ($F(1,33) = 5.84$, $p = 0.02$, $\eta_p^2 = 0.15$). While performance for the two tables differed in the yellow living room (CI = [4.86, 10.85] $p < 0.001$) they did not differ for the blue living room (CI = [-3.2, 5.19], $p = 0.58$). Altogether, these findings indicate that there were no consistent accuracy differences across targets, anchors, or rooms.

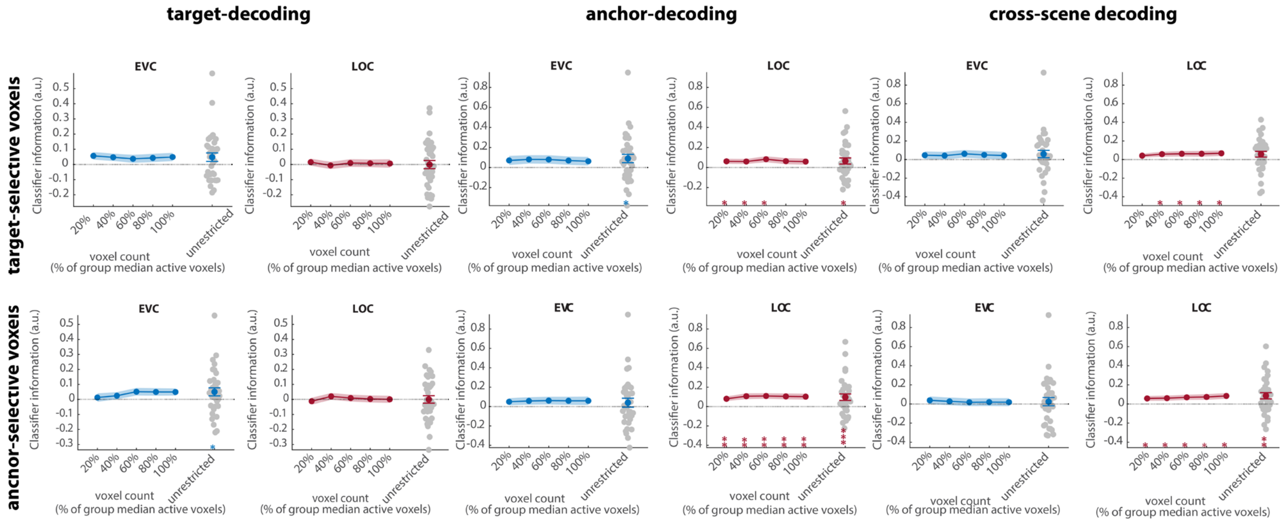

Fig. S1.

Decoding results shown separately for target and anchor-selective voxels. Decoding in EVC and LOC for all selective voxels of individual participants (unrestricted-ROI) and sub-ROIs of different sizes. Grey dots show decoding for individual participants in the largest (unrestricted) ROI. All error bars are SEM. *, $p<0.05$; **, $p<0.01$

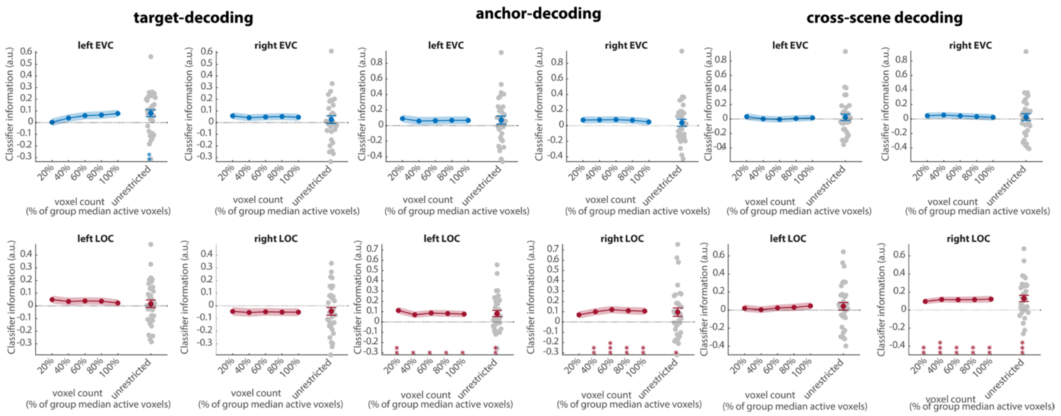

Fig. S2.

Decoding results shown separately for left and right hemisphere. Decoding in EVC and LOC, for all selective voxels of individual participants (unrestricted-ROI), and sub-ROIs of different sizes. Grey dots show decoding for individual participants in the largest (all) ROI. All error bars are SEM. *, $p<0.05$; **, $p<0.01$;***, $p<0.001$

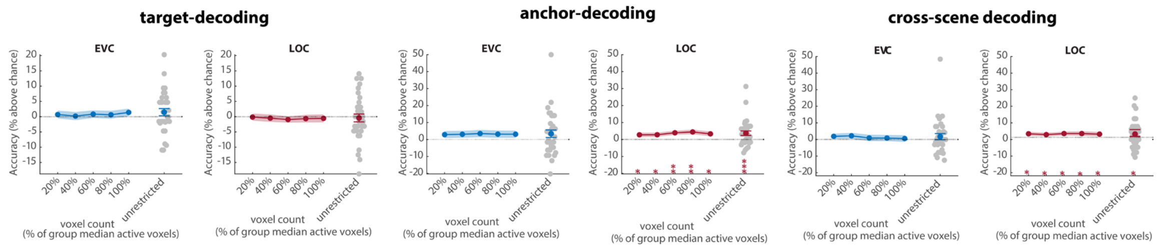

Fig. S3.

Decoding results using decoding accuracy. Decoding in EVC and LOC for all selective voxels of individual participants (unrestricted-ROI), and sub-ROIs of different sizes. Grey dots show decoding for individual participants in the largest (all) ROI. All error bars are SEM. *, p<0.05; **, p<0.01; **, p<0.001

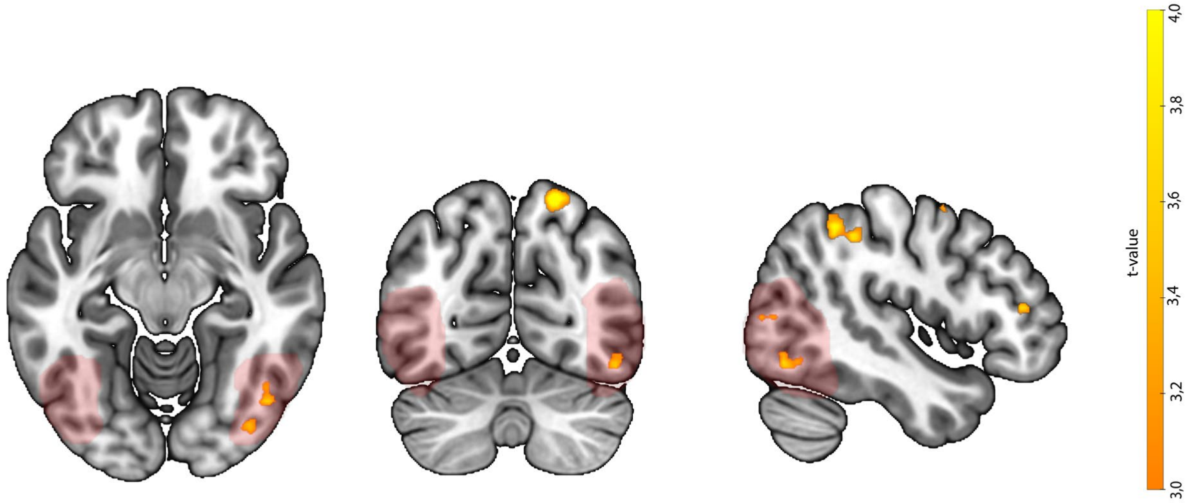

Fig. S4:

Uncorrected whole-brain searchlight (no voxel selection) for decoding the target-associated anchor across scene-contexts. The searchlight shows voxel clusters within the LOC mask (marked in red). Searchlight is thresholded at p = 0.005 (2-sided). Color indicates t-values without threshold-free cluster enhancement (TFCE).
